# Formalizing tenure of Indigenous lands improved forest outcomes in the Atlantic Forest of Brazil

**DOI:** 10.1093/pnasnexus/pgac287

**Published:** 2023-01-26

**Authors:** Rayna Benzeev, Sam Zhang, Marcelo Artur Rauber, Eric A Vance, Peter Newton

**Affiliations:** Department of Environmental Studies, University of Colorado Boulder, Sustainability, Energy and Environment Community, Boulder, CO 80303, USA; Department of Environmental Sciences, Policy, and Management, University of California, Berkeley, CA 94720, USA; Energy and Resources Group, University of California, Berkeley, CA 94720, USA; Department of Applied Mathematics, University of Colorado Boulder, Boulder, CO 80309, USA; Universidade Federal Rural do Rio de Janeiro, Seropédica, RJ 23890-000, Brazil; Department of Applied Mathematics, University of Colorado Boulder, Boulder, CO 80309, USA; Department of Environmental Studies, University of Colorado Boulder, Sustainability, Energy and Environment Community, Boulder, CO 80303, USA

**Keywords:** land tenure, deforestation, Indigenous peoples, Brazil, rights-based conservation

## Abstract

Across the globe, the legal land rights and tenure of many Indigenous peoples are yet to be recognized. A growing body of research demonstrates that tenure of Indigenous lands improves livelihoods and protects forests in addition to inherently recognizing human rights. However, the effect of tenure on environmental outcomes has scarcely been tested in regions with high development pressure, such as those with persisting forest–agriculture conflicts. In this paper, we conduct an event study and a difference-in-differences analysis to estimate the average treatment effect of land tenure on forest cover change for 129 Indigenous lands in the Atlantic Forest of Brazil from 1985 to 2019. We found that forest outcomes in Indigenous lands improved following tenure compared to pretenure and that forest outcomes improved in tenured compared to nontenured lands. We also found that formalized tenure, rather than incomplete tenure, was necessary to improve forest outcomes. Our study is the first rigorous analysis of the effect of tenure on Indigenous lands in the globally important Atlantic Forest biome and contributes to a growing body of literature on the role of rights-based approaches to conservation. The evidence presented in this study may support efforts to secure the legal rights and autonomy of Indigenous peoples.

Significance Statement Large-scale operations such as mining, cattle ranching, and hydroelectric projects are increasingly claiming sizeable tracts of land across the tropics, especially when it is unclear who owns this land. In Brazil’s Atlantic Forest, these activities have displaced local communities, including Indigenous peoples, from lands to which they have a claim. Evidence suggests that granting land tenure to Indigenous peoples can dually improve livelihoods and conserve forests. Our study demonstrates that formalizing tenure for Indigenous lands reduced deforestation and/or increased reforestation in the Atlantic Forest—a region with higher rates of past deforestation than those investigated in many other tropical forest biomes. In doing so, our findings may support an environmental argument to recognize Indigenous peoples with legal land rights.

## Introduction

Indigenous peoples, Afro-descendant populations, and local communities have claims to over half the planet’s land, yet only 10% of the planet is legally recognized with communal land ownership rights (1). Although local communities have long fought to gain autonomy through land rights, many countries have lacked the political interest to strengthen land tenure (hereafter *tenure*)—defined as areas where “communities' rights are legally defined as being unlimited in duration, where they have the legal right to exclude outsiders from using their resources, and they are entitled to due process and compensation in the face of potential extinguishment by the State of some or all of their rights” (2). Encroachment and dispossession by land grabbers, squatters, and extractive industries remains an ongoing challenge for land defenders (3, 4). Some governments and environmental donors are beginning to pay greater attention to the role of tenure as an instrument to tackle environmental and social injustices and climate concerns (5, 6). This interest is supported by emerging evidence suggesting that Indigenous peoples have often been successful in protecting forests and mitigating climate change (7–11).

An important remaining question is whether the broader trend of reduced deforestation and/or increased reforestation in tenured Indigenous lands (ILs) is in part a consequence of remoteness and/or low development pressure. Most studies have evaluated the relationship between tenure and forest outcomes in relatively remote locations (4, 8, 10, 12, 13), or have conducted large-scale analyses where trends in remote areas could have introduced enough variability to reduce the signal of areas with high development pressure (11, 14–16). This is particularly the case if ILs are weighted by size, since remote ILs tend to be larger. Since many ILs are located in rural, densely forested areas with low population densities, it is possible that the combination of land location and limited human impact play a significant role in improved forest conservation (17). There is therefore an important knowledge gap in understanding whether and how tenure of ILs influences forest outcomes in regions with high development pressure and/or regions that do not have any extremely remote ILs. Our study addresses this gap by evaluating the effect of tenure on forest outcomes in ILs in the Atlantic Forest (AF) of Brazil, which is characterized by established infrastructure, economic development, market access, urbanization, contested land rights, higher population densities, and much higher deforestation pressure than other tropical forest biomes.

The primary explanation for the relationship between formalized tenure of ILs and improved forest outcomes (i.e., greater forest conservation or reforestation, and/or reduced deforestation) is that without tenure, land defenders do not have the legal protections to exclude competition for land (4). In the absence of tenure, squatters have often encroached onto territories, deforesting land as a strategy to gain a temporary form of income or to later gain their own use rights or land titles. Insecure tenure may also motivate IL users to deforest at faster rates or to choose more unsustainable production activities, as a consequence of uncertain future access to forest resources (4, 8).

Tenure of ILs is an important issue across the tropics: There has been a broad trend of reduced deforestation in tenured ILs (8, 10, 12, 17, 18) and lower rates of deforestation in ILs compared to other land use types (e.g., counterfactual nonprotected areas) (11, 14, 15, 19, 20). However, at least three studies of ILs have found no effect (4, 13, 21). In addition, in Brazil it is still unclear how different stages of the tenure process contribute to influencing forest outcomes, where stages include the following: stage one: *identification*—identified for anthropological investigation, stage two: *delimitation*—approved by FUNAI—the Brazilian National Indian Foundation, stage three: *declaration*—authorized to be physically demarcated by the Minister of Justice, and stage four: *homologation—*georeferenced boundaries approved by a presidential decree. Understanding the reasons for the variability in the relationship between tenure and forest outcomes—including how this relationship varies by context, why this variation occurs, which stages of the tenure process are important, and to what extent these trends are consistent—will be important information to increase international awareness about the global importance of Indigenous peoples’ land rights and may consequently support the legal cases of contested lands.

**Fig. 1.**
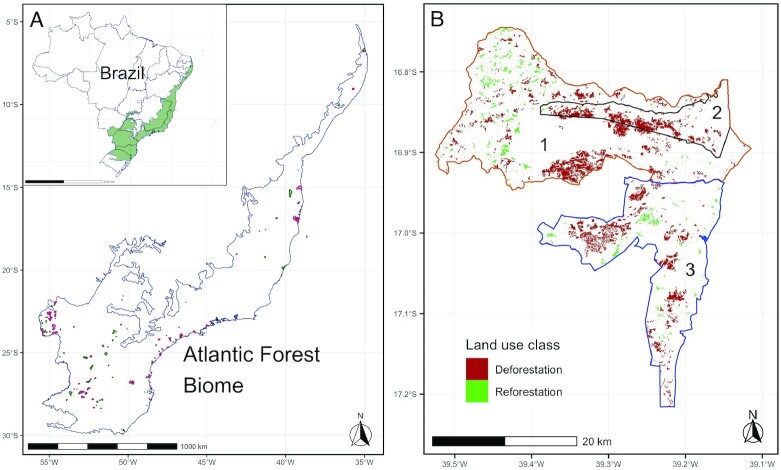
Study site. (A) The locations of ILs with formalized tenure (green) and earlier in the tenure process (red) in the AF biome; inset: the location of the AF biome in Brazil. (B) Forest cover change from 1985 to 2019 in three contiguous ILs in the northeast state of Bahia: Barra Velha (2) has tenure (1991), while Barra Velha do Monte Pascoal (1) and Comexatibá (3) do not (as of March 2022). Forest cover change includes combined changes from 1985 to 2019, which for Barra Velha represents both before and after tenure.

In this paper, we used rigorous causal inference methods to estimate the impact of tenure of ILs on forest cover change in the Brazilian AF biome from 1985 to 2019 (Fig. 1). First, we used an event study (ES) to compare forest cover change before and after 73 ILs gained tenure. Similar to a regression discontinuity in time design, the ES is a flexible model that enables a comparison between before/after effects for panel datasets, including those in which the treatment (i.e., tenure, in this study) is introduced at different points in time (see Supplementary Material). Second, we used staggered difference-in-differences (DID) models to estimate the average treatment effect on the treated (ATT) of land tenure on forest cover change using 129 ILs, of which 77 received land tenure during the observation period. The ILs in the control group were comprised of the 27 territories in stage three of the tenure process and 25 in stage two. The DID models allow for an estimate of the change in forest outcomes for tenured ILs compared to a counterfactual control group, including between tenured and nontenured ILs and before and after tenure. Third, we repeated both analyses after two different stages of Brazil’s tenure (*demarcation*) process to determine whether incomplete tenure and/or formalized tenure caused the observed changes in forest outcomes. The literature on DID designs has advanced rapidly in a short period of time, and these newer approaches are not yet well integrated into the literature on forest conservation. By triangulating across different approaches, this study draws causal conclusions about the effect of tenure on forests. Characterizing the environmental implications of tenure in regions such as the AF, that have persisting legacies of colonization, exploitation, and agricultural conflict, may provide new types of evidence to resolve ongoing court cases and policy decisions for nontenured ILs.

## Results

### Tenure of ILs improved forest outcomes relative to pretenure rates

**Fig. 2.**
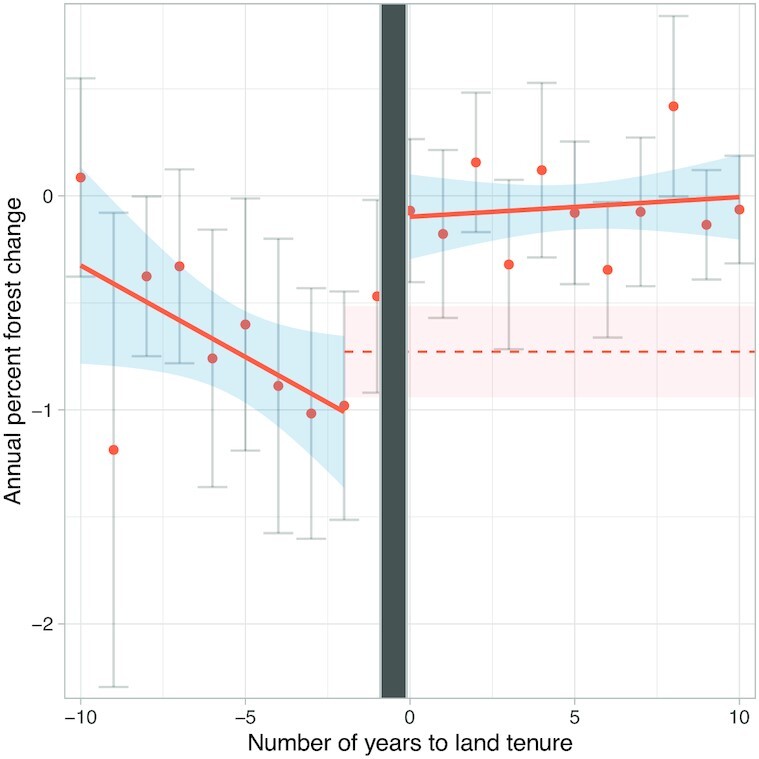
ES analysis displaying trends in *forest cover change* (measured by %) before and after tenure for 78 ILs. Orange points represent binned averages of all ILs in each year in the dataset and 95% CIs (error bars) are included for each point each year. Orange lines represent the average trend and 95% CIs (blue shading) are included for the slope of the mean regression line. The vertical gray bar represents the year of tenure. Due to the anticipation period, the point 1 y prior to tenure was included as part of the year of tenure. The dashed orange line represents the mean of all ILs across all years pretenure to represent an unobserved counterfactual, together with the 95% CI (orange shading).

We conducted an ES (22, 23) as an impact evaluation of *forest cover change* (measured by %), before compared to after tenure for 73 ILs that received tenure. *Forest cover change* represented the net change in the percentage of forest cover of ILs, including both forest gain and forest loss. We found a significant difference between rates of *forest cover change* before compared to after the year of tenure, which indicates reduced deforestation rates and/or increased reforestation following tenure. In our study sample, there was 38,384.1 ha of net deforestation in all ILs during the study period (1985 to 2019). The annual rate of change (averaged across all years) was −0.73% net deforestation before tenure and −0.05% net deforestation after tenure (i.e., a decrease in deforestation after tenure). In terms of area, the annual rate of change was −22.13 ha of deforestation before tenure and −3.32 ha of deforestation after tenure, although these results were not significant (model reported in Table S1). These effects as well as the effects measured in our other models represent the annual (rather than cumulative) rates of forest cover change. Gaining land tenure was therefore associated with an average of a 0.68 percentage point annual decrease in net deforestation (*t* = −5.58, *P* < 0.001) and an average of an 18.8 ha annual decrease in net deforestation (*t* = −4.23, *P* < 0.001). We incorporated a 1-y anticipation period by including the years zero and negative one as the year of tenure (see the “Materials and methods” section and Supplementary Material). We used average pretenure rates of forest cover change as a conservative estimate for the counterfactual, had tenure not occurred (Fig. 2). The general trend was high rates of deforestation prior to tenure with a shift to stabilized deforestation and/or more reforestation following tenure, with rates approaching zero net forest cover change. However, slopes before compared to after tenure were not significantly different, so we could not reject the null hypothesis of significantly similar slopes. Results were negative and significant for the outcome variable *forest cover change* (measured by area, Table S1) and were consistent for several bandwidth estimations, which tested different numbers of years before and after tenure (Table S1).

### Tenure of ILs improved forest outcomes in tenured compared to never-tenured lands and not-yet-tenured lands

**Fig. 3.**
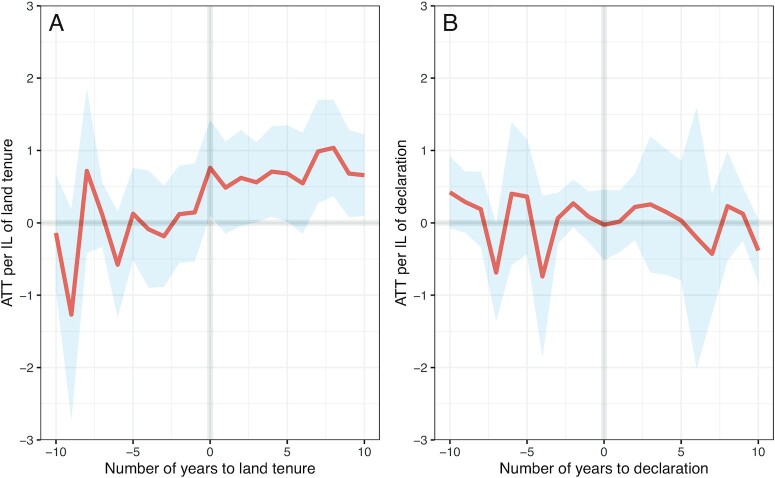
Staggered DID dynamic estimates of the ATT per IL by year on *forest cover change* (measured by %) relative to (A) number of years to formalized tenure (the fourth and final stage of the tenure process), and (B) number of years to declaration (stage three of the tenure process). The red line represents the ATT of tenured ILs and blue shaded area represents uniform 95% CIs around the effect. The model of formalized tenure (A) shows an overall significant result of reduced deforestation and/or increased reforestation, while the model of declaration (B) does not.

We conducted a DID analysis that compared *forest cover change* (measured by %) for ILs with tenure to ILs without tenure, including before and after treatment, to measure counterfactual changes in trends over time. *Forest cover change* was 0.77 percentage points higher each year for ILs with tenure compared to ILs without tenure (i.e., a consistent yearly decrease in deforestation after tenure) [95% CI: (0.22, 1.32), Fig. 3]. This result was robust across three estimators—Callaway and Sant’Anna (2021) (24), de Chaisemartin and D’Haultfœuille (2020) (25), and Sun and Abraham (2021) (26) (see Supplementary Material). This consistency confirms the reliability of our estimates across regressions of causal effects that are reliant on different model assumptions and different estimation techniques (Table 1). The southern region of the AF had stronger trends than the other three regions of Brazil located in the AF (the Northeast, Central-West, and Southeast). In addition, trends were consistent for models with two types of ILs [*Terras Indigenas* (TI) and *Reservas Indigenas* (RIs)—see the “Materials and methods” section]. In contrast to a previous study (10), we excluded RIs from the primary analysis because RIs are not recognized by the Brazilian state and Constitution as ancestral lands but are rather lands that were later granted to Indigenous peoples, often as land that was purchased by the state. We believe that TIs are more policy relevant because many nontenured TIs are awaiting formal tenure, while RIs have already been recognized. Lastly, models were robust to dropping outliers (Table S2). There was no statistically significant result for models with the outcome variable *forest cover change* (measured by area) (Table S2).

**Table 1.** DID estimates (and standard errors) after homologation and after declaration for three estimators.

Estimator	After homologation	After declaration
Callaway and Sant’Anna (2021)	0.767 (0.281)	0.0423 (0.329)
de Chaisemartin and D’Haultfœuille (2020)	0.536 (0.248)	−0.101 (0.172)
Sun and Abraham (2021)	0.705 (0.119)	0.0423 (0.129)

### Formalized tenure resulted in higher forest cover change while no statistically significant trends emerged after declaration

In addition to testing the impact of formalized tenure (*homologation*; the fourth and final stage of the tenure process), we also repeated the DID analysis after the third stage (*declaration—*stage three). In contrast to the statistically significant results found for the 77 territories that received formalized tenure, there was no significant effect for the 27 ILs that had been declared but had not yet received formalized tenure [95% CI: (−0.25, 0.69), Fig. 3]. This finding was also robust to estimator choice (Table 1).

## Discussion

Our analysis reveals that formalizing tenure of ILs resulted in improved forest outcomes across Brazil’s AF. First, the ES demonstrated that tenure of ILs resulted in improvements in forest outcomes compared to pretenure rates. Second, the DID analysis, which was even stronger due to its ability to measure counterfactual trends over time, found a statistically significant difference in forest cover change in that ILs with tenure had improved forest outcomes compared to ILs without tenure, including before and after tenure. Third, repeating the DID analysis after the two final stages of demarcation illustrated that formalized tenure was necessary to improve forest outcomes: improvements occurred following the homologation stage (formalized tenure) but not after the declaration stage. Our study therefore generates robust evidence that tenure of ILs improves forest outcomes in some areas of higher development pressure such as the AF.

Our findings build upon the mixed results of prior studies that evaluated the relationship between forest outcomes and tenure of ILs. Most studies found that tenure of ILs decreased deforestation (8, 10, 11, 17, 18) and studies that analyzed both protected areas and ILs found that the two land designations combined avoided more deforestation than unprotected lands (7, 9, 12, 15). In contrast, some studies found no effect (4, 13, 21), though possible explanations are that effects may not be detectable until later stages of the tenure process than those analyzed (10, 13), or without analyzing longer-term longitudinal datasets. In the context of Brazil, no previous study has explicitly evaluated the impact of tenure on deforestation in the AF biome, though several studies have evaluated this relationship in the Amazon biome (10, 13, 17). In contrast to the Amazon, where deforestation more recently escalated (since the 1970s), deforestation has been prevalent in the AF since the early 16th century and rates were highest in the 19th and 20th centuries (27, 28). The legacy of European conquest and development in the AF meant a higher historical extent of deforestation, as well as a greater degree of market integration, exploitation, and agricultural conflict. As such, although there is a gradient of deforestation pressure across the AF biome, no ILs in the AF are as remote as the set of remote ILs in the Amazon biome. Therefore, our study differs from other studies by having no extremely remote ILs in the study sample.

A number of legal, political, and/or social mechanisms may have caused improvements in forest outcomes with tenure of ILs. First, the current 1988 Brazilian Constitution (hereafter *Constitution*) prohibits the use of tenured lands by non-Indigenous peoples (29). While nontenured ILs in Brazil can be legally occupied by non-Indigenous peoples, often under leasing agreements for mechanized agriculture, tenured ILs cannot be leased according to the Constitution (29). Second, the Federal government is required by law to protect and enforce the rights of tenured ILs from encroachment (10). Third, Indigenous peoples may invest more in their lands when there is certainty that they will be protected (4, 8). See Supplementary Material for details on the primary causes of deforestation over time in ILs in the AF.

Our study additionally demonstrates that tenure improved forest outcomes after formalized tenure, but that there were no significant results after declaration (for the DID analysis). These results are consistent with findings from the Brazilian Amazon that formalized tenure of ILs resulted in reduced deforestation (10), while declaration had no effect on forests (13). Our findings also challenge perceptions of the stages of demarcation in Brazil. While the wording of the 1973 Indian Statute indicates that the recognition of land rights of ILs will not depend on their formal tenure (Article 25) (30, 31), our study suggests that formalized tenure is critical to achieving real-world recognition and improved forest outcomes. Given the relatively small sample size of declared ILs in this study, it would be worthwhile for future research to further investigate the role of different demarcation stages on outcomes for forests and livelihoods.

By demonstrating that tenure of ILs improved forest outcomes in the AF, where a legacy of land dispossession has caused numerous instances of forced migration and deforestation, our study supports the general trend that tenure of ILs improves forest outcomes across heterogeneous contexts. The evidence that tenure of ILs leads to improved environmental outcomes is stronger than the evidence for the effects of tenure on non-ILs. Two-thirds of studies that examined the impact of tenure across a number of land use types have found a positive relationship between tenure and either improved human well-being and/or environmental outcomes (32). However, studies that specifically examined tenure of ILs suggest that rates of deforestation on ILs across the tropics have been comparable to deforestation within protected areas and have been much lower than deforestation on other land use types (11). Our study further contributes to this literature.

Our results suggest considerable potential for reduced deforestation and/or recovery of forests in ILs in the AF if lands that do not have formalized tenure complete the final stage of the tenure process. Indeed, the southern region of the biome had the highest number of nontenured ILs, the highest number of ILs with land conflicts, and a stronger relationship between tenure and forest outcomes than the three other regions of the AF (ATT of 0.964 rather than 0.821) (see Supplementary Material). Currently, however, demarcation appears to have stalled for many ILs: of the 726 ILs in Brazil, 122 remain under study (stage one), 44 are delimited (stage two), and 74 are declared (stage three), but not homologated by presidential decree (stage four) (29). In fact, in our study sample only one IL had been demarcated since 2012. To the extent that these delays are associated with proposed legislative changes by political lobbies and the pro-agribusiness Brazilian Congress (29, 33, 34), renewed demarcation of ILs may be facilitated by (1) upholding the Constitution’s recognition of all Indigenous peoples in Brazil as “legitimate original landholders” (29, 34, 35), and (2) ensuring that FUNAI has the resources and political support to protect, demarcate, and monitor land rights of ILs (29, 33, 36). Proposed reinterpretations of the Constitution, such as the 2021 *marco temporal* case, which suggests restricting tenure only to Indigenous peoples who had been inhabiting their lands on the exact date when the Constitution was established (34, 36–38), may face stronger opposition if these objectives are realized.

Our study was limited in its ability to highlight the heterogeneity between different ILs and in its measurement of one outcome variable. The variance in our data and models do speak to the different land-use trajectories across ILs: for example, we found that different ILs had substantial variations in forest cover change year to year, with many ILs following divergent zigzagging trajectories over time, which may have been partially due to the use of shifting cultivation practices in some ILs. However, our analysis was not able to reveal anything about the underlying processes, decisions, institutions, and resistance efforts within different ILs. Studies that apply methodologies and analyses to explore this variation, such as in-depth ethnographic or other qualitative research, may better reflect the diverse relations that individuals and groups living within ILs have with the land. Furthermore, our quantitative study considered a single set of environmental outcomes (i.e., forest cover change, as the aggregate of forest gain and loss) but did not include any analyses of the many important social and political outcomes associated with tenure of ILs. These other outcomes, including rates of violence, murders of Indigenous leaders, perceived discrimination, and number of evictions, or cultural variables such as identity, maintenance of original language, and participation in traditional livelihood activities, are very important to study. An analysis of multiple outcomes could offer insights into the multiplicity of values gained from tenure and how these outcomes covary. However, absent spatially explicit, annual data on any of these social or cultural variables, such analyses remain challenging to conduct at scale. Similar to other studies (10), our data did not enable us to measure leakage. We consider spatial leakage (i.e., reduced deforestation in ILs could be offset by increased deforestation elsewhere) unlikely, given the distinct governance regimes and populations involved in ILs. We also consider temporal leakage (i.e., increased deforestation in ILs by Indigenous peoples in anticipation of tenure) also to be unlikely, since deforestation by Indigenous peoples is not prohibited by tenure.

Our results present robust evidence that ILs with tenure reduce deforestation and/or improve reforestation, even in a context with strong pressures from economic development, colonization, land disputes, and deforestation. Our study contributes to an emerging body of evidence suggesting that rights-based policy for ILs can improve environmental outcomes. Legally recognizing Indigenous peoples’ land rights is one approach that enables Indigenous peoples to reclaim territorial autonomy and gain self-determination rights, which may support efforts to address longstanding human rights violations, land grabs, biodiversity loss, and climate change.

## Materials and methods

### Data

The panel dataset included the independent variable *tenure*, defined as the date of the homologation decree as reported by FUNAI (the Brazilian National Indian Foundation), in accordance with the Federal Constitution (CF/88, Law 6001/73–Indian Statute, Decree No.1775/96). The dataset included most ILs located in the AF—129 ILs (TIs) for the primary analysis (reported above and in Table S3) and an additional 25 Indigenous reserves (RIs) for a secondary analysis (reported in the Supplementary Material). Data were sourced from the most recently available document from the Ministry of Justice and Public Safety at FUNAI (SEI No. 2208867), which was last updated in October 2019 and acquired from the Fala.BR data acquisition platform (39, 40). ILs were selected by intersecting the AF biome boundary (41) with the geospatial locations of all ILs in Brazil (39). The 44 ILs in the AF that were “under study” were not included since these lands did not have official and/or publicly available geospatial boundaries. As such, our sample included a large majority of those ILs that had started the tenure process, with few exceptions (see Supplementary Material). Missing data from the 2019 FUNAI dataset were also acquired from Fala.BR (39) (see Supplementary Material). Data were aggregated to the year of tenure (rather than to the month or day). *Tenure* was formatted as a binary variable representing tenure formalization for each IL each year, which included the year of tenure.

The dependent variable was *forest cover change* (measured by %) and was reported as the ATT. *Forest cover change* was defined as a change in natural forest formation classification in the fifth collection of the MapBiomas Project (42), which included primary forest, secondary forest, and mixed agroforests, but excluded savannah, mangroves, and forest plantations. This definition was consistent with other studies that have used MapBiomas data (43). We selected *forest cover change* (measured by %) rather than *forest cover change* (measured by area) because it: (a) is a more politically relevant variable given that policy for tenure occurs by IL rather than by land area, (b) avoids biasing results toward larger ILs, and (c) has precedent in previous studies (8, 10). Findings for *forest cover change* (measured by area) are reported in the “Results” section and Supplementary Material (Table S2). The ATT was the average treatment effect for the subpopulation of ILs that received treatment (land tenure or declaration). Annual forest cover data spreadsheets from 1985 to 2019 were sourced from the online interactive platform of MapBiomas (42). Data were derived from Landsat 30*m* × 30*m* pixel imagery and measured in hectares. Data were estimated by MapBiomas using pixel-per-pixel image-processing algorithms in Google Earth Engine for preprocessing and normalization, a random forest classifier to map land use classes, and cloud-shadow masks algorithms to overcome cloudiness limitations (44). *Forest cover change* (measured by area) was calculated by subtracting forest cover from the previous year by forest cover from the current year, to obtain a dataset representing change from 1986 to 2019. *Forest cover change* (measured by %) was calculated as *forest cover change* (measured by area) divided by IL size multiplied by 100.

### Analysis

#### Event study

We selected the ES approach to compare trends before and after the event of tenure using a panel dataset. Any IL that received tenure before 1986, during the year 1986, or during the year 2019 were excluded because the ES required a measurement of a change before and after tenure. As such, the sample size for the ES (*n* = 73) was slightly smaller than the sample for the DID (*n* = 77). We centered the data with zero as the year of tenure by subtracting the year of tenure from the current year for each entry. Number of years to tenure represented the variable determining treatment and zero (year of tenure) represented the cutpoint. We fit a local linear regression as recommended by Gelman and Imbens (2019) (45) and modeled different slopes on either side of the cutpoint

$$
\begin{eqnarray*}
Y\; = \;\alpha + \;\tau 1\left\{ {t \ge 0} \right\} + \;{\beta _1}t + {\beta _2}1\left\{ {t \ge 0} \right\}t + \varepsilon ,
\end{eqnarray*}
$$

where *t* is the number of years since tenure. Since tenure occurred at different points in time, we were able to control for unmeasured time-variant factors, which depends on the assumption that it is unlikely for there to be another event that is also correlated with the timing of tenure across ILs. We selected the range of years to use in the ES as up to 10 y before and after tenure, but we also ran model specifications for several bandwidth estimations (± 5, 15, and 20) to test robustness (Table S1). We additionally ran models for four regions of the biome and without the three primary outlier ILs (Table S1). We expected that trends in the southern region of the AF may have been different than other regions of the AF due to the higher number of nontenured ILs relative to other regions and the high level of land conflicts in this region. Model assumptions are described in the Supplementary Material.

Given the possibility of an anticipation period (24, 46, 47), we examined the data for an anticipation effect by running the ES with anticipation periods of 0 or 1 y. The presence of a 1-y anticipation period led us to regard the datapoint 1 y before tenure as part of the cutpoint rather than as a datapoint in the regression of pretenure trends. This anticipation was likely caused by a combination of (1) the lag that commonly occurs between the announcement of tenure and the date of tenure formalization, and (2) aggregating *tenure* to year rather than to month or day. The announcement of tenure may occur when Indigenous peoples inquire about the status of the tenure process with FUNAI employees before homologation. Alternatively, the demarcation of physical land boundaries, which occurs sometime between the declaration and homologation stages, may be a signal that homologation is approaching. However, data on the dates of demarcation of physical land boundaries are not available (as of March 2022).

#### Staggered DID

The staggered DID design was selected based on the methods of similar studies with multiple time periods, variation in treatment timing, and parallel trends assumptions after conditioning on covariates (48, 49). We selected this approach instead of a regression discontinuity or a regression discontinuity in time design given that (1) we were not using geography as the continuous (running) variable, (2) the effects were observed not at a single cross-sectional time point but rather across a period of time, (3) this period of time included not just the immediate short-term effects but also data farther from the cutpoint, (4) advancements in DID methods enable us to perform causal inferences on this balanced panel data (50, 51). ILs received tenure at different points in time, ILs did not lose tenure status after being formalized (Fig. S1), and we observed a balanced panel of data where each IL was observed in every year. As such, we used the doubly robust staggered DID estimator from Callaway and Sant’Anna (2021), with tenured lands as the treatment group and nontenured lands as the control. Our basic unit of analysis was a single unweighted IL, since land titling occurred at the IL level. We controlled for the size of each IL in log-hectares and all inference procedures used clustered bootstrapped standard errors at the IL level. The approach of Callaway and Sant’Anna estimated treatment effects for each pair of groups *g* and times *t*, where *g* refers to the year that a unit received treatment:

$$
\begin{eqnarray*}
ATT\;\left( {g,t} \right)\, = \,{\mathbb{E}}\left[ {{Y_t}\left( g \right) - {Y_t}\left( g \right)\left| {\;{G_g} = 1} \right.} \right].
\end{eqnarray*}
$$

The dynamic and overall effects were computed by aggregating group-time treatment effects. For the dynamic effect, we computed a weighted average of group-time treatment effects with respect to years elapsed after treatment, *e* = *t*− *g*:

$$
\begin{eqnarray*}
{\theta _{es}}\;\left( e \right) = \mathop \sum \limits_{g \in \mathcal{G}} 1\left\{ {g + e \le \mathcal{T}} \right\}P(G = g|G + e\; \le \mathcal{T})ATT\left( {g,\;g + e} \right),
\end{eqnarray*}
$$

where *G* is the year when a unit was treated, $\mathcal{G}$ is the set of all years when at least one unit received treatment, and $\mathcal{T}$ is the most recent year in the data. We restricted our analysis to the 10 y before and after treatment, and we generated uniform 95% CIs using 1000 bootstrap replications. For the overall effect, we averaged all group-time treatment effects weighted by group size (referred to by Callaway and Sant’Anna as the “simple aggregation”).

We tested whether incomplete tenure and/or formalized tenure caused changes in forest outcomes by testing the DID after an earlier stage of the tenure process (declaration), which we restricted to ILs that never received tenure to avoid the confounding effects of later receiving tenure. There were smaller sample sizes for this declaration analysis because of less widely available data and our choice to only include ILs that never received tenure. We additionally ran models that separated the biome into two regions, dropped the three primary outlier ILs, and used alternate estimators (see Supplementary Material). The regional analyses were originally split according to the four regions of Brazil that exist within the AF biome, but the small sample sizes in some regions led us to split the biome by “south” and “not south” for the DID analysis, since the southern region had the largest sample (Table S3). Model assumptions are described in Supplementary Material.

## Supplementary Material

pgac287_Supplemental_File
