# Supplementary material for: Formalizing tenure of Indigenous lands improved forest outcomes in the Atlantic Forest of Brazil
Source: PNAS Nexus. 2023 Jan 26;2(1):pgac287. doi: 10.1093/pnasnexus/pgac287 (PMC9879837; doi:10.1093/pnasnexus/pgac287)
Supplement: pgac287_Supplemental_File [file pgac287_supplemental_file.docx]

**Supplementary Information for**

Formalizing tenure of Indigenous lands improved forest outcomes in the Atlantic Forest of Brazil

Rayna Benzeev, Sam Zhang, Marcelo Artur Rauber, Eric A. Vance, Peter Newton

**Corresponding Author:** Rayna Benzeev

**Email:**  [Rayna.benzeev@colorado.edu](mailto:Rayna.benzeev@colorado.edu)

**This PDF file includes:**

Supplementary text

Figure S1

Tables S1 to S2

SI References

Supplementary Information Text

**Possible explanations for deforestation trends**

The primary explanations for improvements in forest change with land tenure are that 1) tenure insecurity incentivizes encroachment by squatters, and 2) encroachment increases deforestation by Indigenous Peoples due to insecure future access to land (1–3). Five additional related factors may have explained changes in rates of deforestation in the AF context. First, Indigenous Lands (ILs) without formalized tenure may have had a stronger legacy of state-led colonization and assimilation programs, which were used as a rationale to encroach onto ILs (4, 5). These public policies directly promoted deforestation, aiming to increase logging, establish wheat, maize, and soy agriculture, and create more pasture for livestock (6). The Brazilian National Indian Foundation (FUNAI) and the Indian Protection Service (SPI) were involved in these public policies (7), such as for the logging of Araucária trees in the 1960s to 1980s, which was of significant cultural importance to the Kaingang peoples in the states of Santa Catarina and Rio Grande do Sul (8). Although public policies incentivizing deforestation and encroachment gradually reduced after the 1980s, land conflicts persist today, making it difficult for Indigenous Peoples to reclaim these lands. Second, the process of illegally renting land in ILs may have increased deforestation of non-tenured lands (9). Although illegally renting ILs was more prevalent before 2008 and has since slowed, recently, the Bolsonaro administration has attempted to make it legal to rent land in ILs (10). Given that attempts to legalize renting land increases the number of actors attempting to make claims to disputed lands, this process is likely to exacerbate and/or complexify land conflicts. Third, during former President Lula’s administration (2003 to 2010), more fines were established for deforestation alongside better monitoring using satellite imagery, which could have slowed deforestation during this time (11). Fourth, changes in Indigenous leadership can account for changes of land use within ILs. For example, Indigenous leaders have been involved in several court cases and investigations by the Federal Police, including arrests of Indigenous leaders on suspicion of allowing farmers to use large swaths of land for soybean production, resulting in high rates of deforestation and heightened land conflicts (12, 13). Fifth, in some cases, Indigenous Peoples have been completely driven out of sections of their lands. For example, the demarcation of Barra Velha in 1991 enabled many Pataxó in the extreme south of Bahia to return to a section of their original land, after being dispersed elsewhere. However land conflicts across other nearby non-tenured ILs are still prevalent, where much land is occupied by non-Indigenous people (14). The changes in policy and in the political landscape during our study period likely resulted in different amounts of deforestation pressure over time. However, because tenure was formalized at various points in time for different ILs, our analyses demonstrate that the influence of tenure on forests existed irrespective of any individual event or year.

**Alternate Model Specifications**

In addition to the primary model, which we reported in the main manuscript, we reported several other model variations (Tables S1 and S2). These included 1) a dataset that included both *Terras Indigenas* (TIs) and *Reservas Indigenas* (RIs) (see Results), 2) *forest change* (measured by area) as the outcome variable, 3) an analysis without the three primary outliers (each of which had both the three highest local maximums and three lowest minimums), and 4) a regional analysis for each of the four regions of Brazil located in the AF (the Northeast, Central-West, Southeast, and South; see Table S3), and 5) different bandwidths, i.e., considering various windows 5 years before and after tenure, 15 years, and 20 years (for the ES only) (Table S1). For the event study (ES), all model specifications were statistically significant with negative estimates and statistics, with the exception of the southeast region, which was not statistically significant (Table S1). For the difference-in-difference (DID) analysis, all models with the outcome variable *forest change* (measured by percent) were statistically significant, which corresponded to the outcome variable used in similar studies (15, 16), while models for outcome variable *forest change* (measured by area) were not statistically significant (Table S2). We expect that *forest change* (measured by area) models may not have been significant because this outcome variable was biased towards larger ILs, and many of the larger ILs in the AF tended to be non-tenured (Fig. 1). Since larger ILs were likely to have greater amounts of forest change (in either direction) than smaller ILs, datasets with a greater number of large ILs without tenure may have changed the nature of the results in comparison to the outcome variable *forest change* (measured by percent). All model variations for each outcome variable had similar estimates of ATT, indicating robustness of this method. For the regional analysis, we found that trends in the south were stronger than trends in the other regions combined (see Methods).

To address the concern that never-tenured ILs could be systematically different from eventually-tenured ILs, we ran the analysis dropping all never-tenured lLs, using only the not-yet-tenured lands as controls (N=2618 observations over 77 ILs). We found that *forest change* was 0.66 percentage points higher among tenured lands compared to not-yet tenured lands (95% CI: [0.06, 1.27]). In this analysis, we found violations of the overlap assumption, and thus we followed the recommendation of Callaway and Sant’Anna in using first-step regression rather than doubly-robust estimation to compute group-time average treatment effects. Moreover, since the not-yet-treated units were not available for all ILs, the overall average treatment effect was generated omitting years after 2010.

**Model Assumptions**

*Event study*

**No systematic changes over time except for the treatment.** For a description of the possible causes of deforestation over time see the section titled “Possible explanations for deforestation trends” above. Since tenure was formalized at many different points in time for different ILs, individual events at single points in time were unlikely to explain the overall trend.

*Difference-in-difference*

**Irreversibility of treatment.** ILs were recognized with formal tenure before the start of the study period up until 2016 and did not lose tenure status after gaining tenure (Fig. S1).

**Random sampling.** There were no systematic differences between ILs with and without tenure. ILs with and without tenure were dispersed across space (Fig. 1), spanning different states and regions of the AF biome. However, it is possible that ILs without tenure have been located on more desirable agricultural land, which has caused increased land conflicts over time and subsequent delays in demarcation.

**Limited treatment anticipation.** The ES analysis led us to expect a one-year anticipation period, but we had no reason to expect relevant anticipation of the treatment prior to one year. The analysis of ILs receiving declared status further reinforced this point.

**Conditional parallel trends.** We use two specific conditional parallel trends assumptions in the estimator of Callaway and Sant’Anna (2021) (17): one assumption for non-tenured ILs and one assumption for non-tenured ILs that will later be tenured. The assumption was that after controlling for covariates (the log-size of the ILs), the non-tenured ILs followed the same path as the tenured ILs would have followed had they never received the counterfactual of tenure.

**Overlap.** We assume sufficient covariate overlap such that the propensity score estimates are bounded away from 1. Specifically, for each $t\in\left\{ 2,\ldots, \mathcal{T} \right\},g\in\mathcal{G}$, there exist some $\varepsilon>0$ such that $P\left( G_{g}=1 \right)>\varepsilon and p_{g,t}\left( X \right)>1-\varepsilon a.s.$, where *p* is the generalized propensity score (17).

**Alternate estimators**

We checked the robustness of our results to the choice of estimator by using two other methods appropriate for staggered DID that have different assumptions than Callaway and Sant’Anna (CS) (17)—de Chaisemartin and D'Haultfœuille (dCDH) (18) and Sun and Abraham (SA) (19). The dCDH estimator tests for instantaneous effects of treatment between years $t$ and $t+1$, allows for heterogeneity in both groups and time, and allows for reversibility of treatment with an additional parallel trends assumption (which is not necessary in our study since ILs that receive tenure never lose tenure). The dCDH estimator therefore employs a slightly weaker parallel trends assumption for our study since only the instantaneous treatment effect is necessary. With a binary treatment and a staggered adoption, the SA estimator is identical to the estimator of Imai and Kim (2021) (20). Given that there was no simple way to incorporate the anticipation period in the dCDH estimator, we estimated the effect without accounting for anticipation, leading to a more conservative estimate. Furthermore, dCDH did not provide a way to control for covariates, so we did not control for the size of the territory. Since dCDH estimates the instantaneous treatment effect, we used all data for the analysis before and after declaration, rather than only the ILs that were declared but not tenured. We used analytical standard errors for SA and bootstrap standard errors using 100 bootstrap replications for dCDH.

The SA estimator uses a similar set of assumptions to CS, except with the crucial difference that it assumes homogeneity across groups. Specifically, treatment effects can be dynamic over time but different groups must experience the same treatment profiles conditional on covariates. Moreover, the SA estimator can only use the never-treated and last-treated cohort as controls, and it requires the selection of two years as reference periods. We chose years -1 and -2 as our reference periods and used the same reference points for the analysis before and after declaration.

**Omissions and additions to the dataset**

**Mapbiomas.** Following the collection of MapBiomas data, we omitted one IL, Muã Mimatxi (Fazenda Modelo Diniz), as this data was absent from MapBiomas (as of 2021).

**FUNAI.** There were several missing data in the 2019 FUNAI dataset that we acquired by emailing FUNAI at [sic@funai.gov.br](mailto:sic@funai.gov.br). First, FUNAI provided us with data for two ILs that existed as entries in the shapefile dataset but not in the tenure dataset (Barragem Norte and Tekoa Gwyra Pepo). Second, they provided us with the year of homologation (or establishment for RIs) for 11 ILs that were present in the dataset but had blank entries (Águas Claras, Amaral/Tekoá Kuriy, Cachoeira dos Inácios, Cachoeirinha (Terena), Canelinha, Fazenda Boa Vista, Geripancó (Jeripancó), Hãm Yîxux, Morro da Palha, Mundo Verde/Cachoeirinha, and Riozinho). For three RIs, FUNAI was unable to provide the year of establishment (Dourados, Takuari, Tekohá Añetete, and Tekohá Itamarã), and therefore we omitted these from the dataset. In addition, we omitted three duplicates from the FUNAI shapefile dataset (Jaraguá, Kariri – Xocó, and Wassu-Cocal), but maintained ILs with duplicate names that represented multiple ILs managed by the same peoples (Boa Vista Sertão do Promirim, Jaraguá, Kariri-Xocó, Nonoai, Pindoty, Toldo Chimbangue, Votouro, and Xapecó). To convert numerical data from Brazilian notation to US notation, we replaced all commas with periods to represent decimal values. Last, although some RIs were labeled with the phrase “Encaminhada RI” on the spreadsheet, some were not. As such, we cross-checked the labels for ILs by searching for the category listed on the Socio-Environmental Institute (ISA) website (21) and from a list of Brazil’s presidential decrees.

Data on declaration was not available for ILs declared before 1991, as a different tenure formalization process was in place.


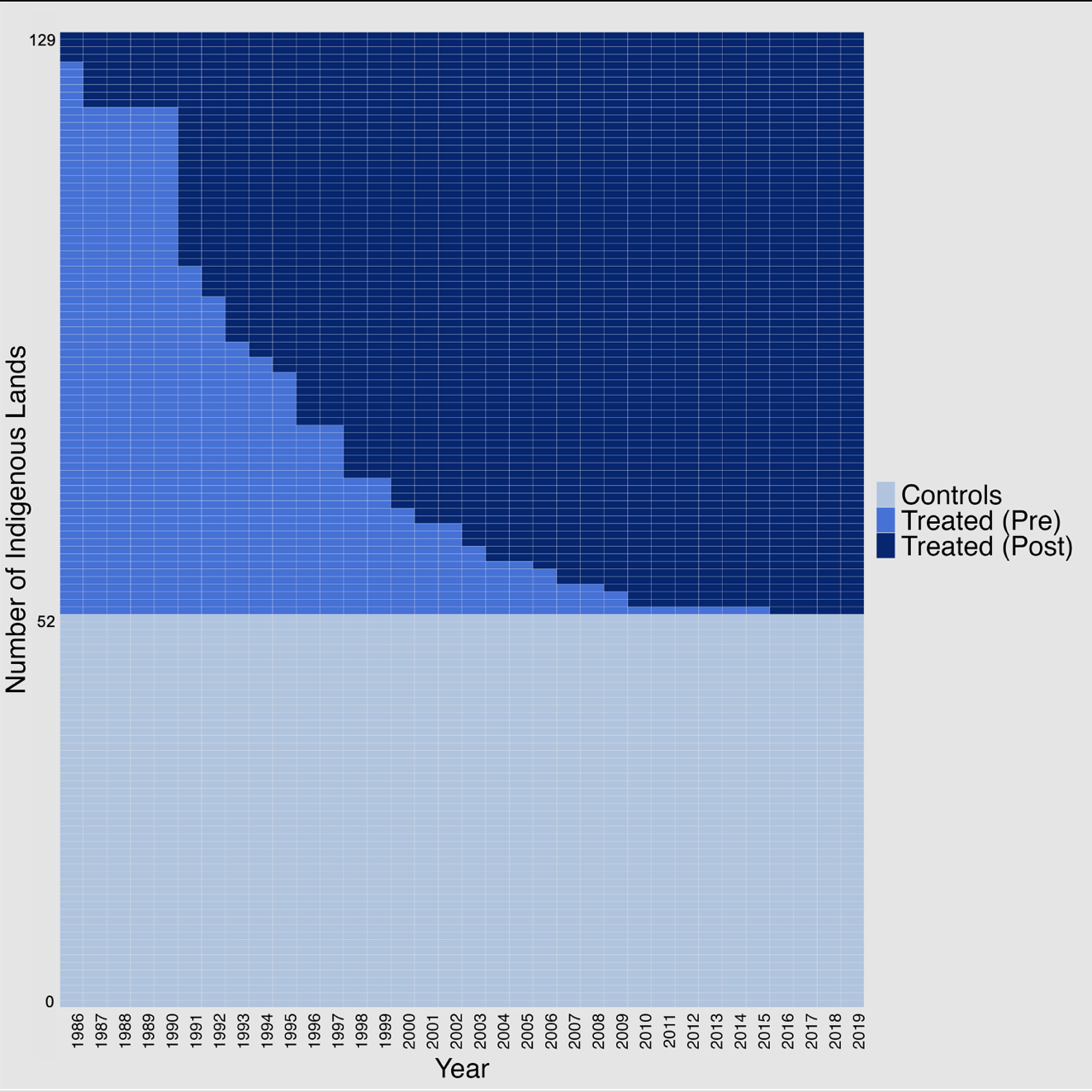


Fig. S1. Staggered study design, including number of Indigenous lands (ILs) with and without tenure per year of the study. ‘Controls’ represent ILs never with tenure, ‘Treated (Pre)’ represents ILs without tenure that later were tenured, and ‘Treated (Post)’ represents ILs with tenure. The year when the largest number of ILs gained formal tenure was 1991, as this was the year when a presidential decree was established that solidified a new process for recognizing ILs, following the establishment of the 1988 constitution. Before 1991, a different process for formalizing ILs was in place, and the decision to formalize ILs was controlled by the military.

Table S1. Event study (ES) estimates (and standard errors) for 11 model specifications.

| **Model** | **Estimate** | **P-value** |
| --- | --- | --- |
| Primary model | -0.676 (0.121) | <0.001 |
| Combined TIs and RIs | -0.650 (0.115) | <0.001 |
| Area of forest change | -18.8 (4.44) | <0.001 |
| Without outliers | -0.676 (0.121) | <0.001 |
| Region: northeast | -1.25 (0.454) | <0.007 |
| Region: central-west | -1.51 (0.476) | <0.002 |
| Region: southeast | 0.00593 (0.163) | 0.971 |
| Region: south | -0.718 (0.126) | <0.001 |
| Bandwidth: -5 to 5 | -0.810 (0.171) | <0.001 |
| Bandwidth: -15 to 15 | -0.536 (0.102) | <0.001 |
| Bandwidth: -20 to 20 | -0.529 (0.0953) | <0.001 |

Table S2. Difference-in-difference (DID) estimates (and standard errors) for ten model specifications.

| **Dependent variable** | **Stage of demarcation analyzed** | **Dropped outliers** | **Terras Indigenas (TI) / Reservas Indigenas (RI)** | **Region** | **ATT** | **P-value** |
| --- | --- | --- | --- | --- | --- | --- |
| Forest change (percent) | Formalized tenure | No | TIs only | All | 0.767 (0.290) | 0.004 |
| Forest change (percent) | Formalized tenure | No | TIs and RIs | All | 0.780 (0.250) | <0.001 |
| Forest change (percent) | Formalized tenure | Yes | TIs only | All | 0.787 (0.287) | 0.003 |
| Forest change (percent) | Formalized tenure | Yes | TIs and RIs | All | 0.798 (0.239) | <0.001 |
| Forest change (percent) | Formalized tenure | No | TIs only | South | 0.964 (0.339) | 0.002 |
| Forest change (percent) | Formalized tenure | No | TIs only | All except South | 0.821 (0.470) | 0.040 |
| Forest change (percent) | Declaration | No | TIs only | All | 0.042 (0.329) | 0.449 |
| Forest change (area) | Formalized tenure | No | TIs only | All | 0.441 (0.445) | 0.161 |
| Forest change (area) | Formalized tenure | No | TIs and RIs | All | 0.412 (0.395) | 0.148 |
| Forest change (area) | Declaration | No | TIs only | All | -0.005 (0.815) | 0.502 |

**Table S3.** Indigenous lands included in the study. Data were sourced from FUNAI (the Brazilian National Indian Foundation).

| Indigenous land | State | Region | Size (ha) | Stage | Year of declaration | Year of tenure |
| --- | --- | --- | --- | --- | --- | --- |
| Comboios | Espírito Santo | Southeast | 3,908.66 | Tenured | 2007 | 2010 |
| Coroa Vermelha | Bahia | Northeast | 1,502.75 | Tenured | 1997 | 2000 |
| Faxinal | Paraná | South | 1,874.76 | Tenured | No data | 1991 |
| Fazenda Guarani | Minas Gerais | Southeast | 3,410.14 | Tenured | No data | 1991 |
| Guaimbé | Mato Grosso do Sul | Center-West | 725.77 | Tenured | No data | 1984 |
| Guarani Araponga | Rio de Janeiro | Southeast | 215.23 | Tenured | 1994 | 1995 |
| Guarani Barra do Ouro | Rio Grande do Sul | South | 2,260.47 | Tenured | 1998 | 2001 |
| Guarani da Barragem | São Paulo | Southeast | 29.86 | Tenured | No data | 1987 |
| Guarani de Bracuí | Rio de Janeiro | Southeast | 2,125.26 | Tenured | 1994 | 1995 |
| Guarani do Aguapeu | São Paulo | Southeast | 4,427.63 | Tenured | 1994 | 1998 |
| Guarani Votouro | Rio Grande do Sul | South | 747.25 | Tenured | No data | 1998 |
| Guarita | Rio Grande do Sul | South | 23,238.16 | Tenured | No data | 1991 |
| Guasuti | Mato Grosso do Sul | Center-West | 885.55 | Tenured | 1991 | 1992 |
| Ibirama | Santa Catarina | South | 14,011.63 | Tenured | No data | 1996 |
| Icatu | São Paulo | Southeast | 291.03 | Tenured | No data | 1991 |
| Amambai | Mato Grosso do Sul | Center-West | 2,427.72 | Tenured | No data | 1991 |
| Ilha da Cotinga | Paraná | South | 1,734.52 | Tenured | 1992 | 1994 |
| Imbiriba | Bahia | Northeast | 391.15 | Tenured | 2004 | 2007 |
| Inhacorá | Rio Grande do Sul | South | 2,796.05 | Tenured | No data | 1991 |
| Ivaí | Paraná | South | 7,302.69 | Tenured | No data | 1991 |
| Jacaré de São Domingos | Paraíba | Northeast | 5,017.97 | Tenured | 1992 | 1993 |
| Jaguapiré | Mato Grosso do Sul | Center-West | 2,301.32 | Tenured | 1992 | 1992 |
| Jaguari | Mato Grosso do Sul | Center-West | 411.31 | Tenured | 1991 | 1992 |
| Jaraguá | São Paulo | Southeast | 1.52 | Tenured | No data | 1987 |
| Jarará | Mato Grosso do Sul | Center-West | 506.72 | Tenured | 1992 | 1993 |
| Kaingang de Iraí | Rio Grande do Sul | South | 271.86 | Tenured | 1992 | 1993 |
| Kariri-Xocó | Alagoas | Northeast | 700.23 | Tenured | No data | 1993 |
| Krenak | Minas Gerais | Southeast | 4,005.4 | Tenured | 1992 | 2001 |
| Krukutu | São Paulo | Southeast | 29.27 | Tenured | No data | 1987 |
| Laranjinha | Paraná | South | 288.97 | Tenured | No data | 1996 |
| Ligeiro | Rio Grande do Sul | South | 4,581.66 | Tenured | No data | 1991 |
| Mata Medonha | Bahia | Northeast | 640.53 | Tenured | 1993 | 1996 |
| Maxacali | Minas Gerais | Southeast | 5,286.1 | Tenured | 1993 | 1996 |
| Mbiguaçu | Santa Catarina | South | 60.84 | Tenured | 2000 | 2003 |
| Monte Caseros | Rio Grande do Sul | South | 1,089.82 | Tenured | 1996 | 1998 |
| Nonoai/Rio da Várzea | Rio Grande do Sul | South | 16,119.05 | Tenured | 1998 | 2003 |
| Palmas | Paraná/Santa Catarina | South | 3,738.81 | Tenured | 2004 | 2007 |
| Panambizinho | Mato Grosso do Sul | Center-West | 1,280.74 | Tenured | 1995 | 2004 |
| Parati-Mirim | Rio de Janeiro | Southeast | 94.16 | Tenured | 1994 | 1996 |
| Peruíbe | São Paulo | Southeast | 477.81 | Tenured | No data | 1994 |
| Araribá | São Paulo | Southeast | 1,931.6 | Tenured | No data | 1991 |
| Piaçaguera | São Paulo | Southeast | 2,744.2 | Tenured | 2011 | 2016 |
| Pinhal | Santa Catarina | South | 880.51 | Tenured | 1994 | 1998 |
| Pirajuí | Mato Grosso do Sul | Center-West | 2,132.42 | Tenured | No data | 1986 |
| Porto Lindo | Mato Grosso do Sul | Center-West | 1,663.96 | Tenured | No data | 1991 |
| Potiguara | Paraíba | Northeast | 21,312.33 | Tenured | No data | 1991 |
| Queimadas | Paraná | South | 3,004.75 | Tenured | No data | 1996 |
| Rancho Jacaré | Mato Grosso do Sul | Center-West | 767.27 | Tenured | No data | 1984 |
| Guarani do Ribeirão Silveira | São Paulo | Southeast | 8,465.94 | Tenured | No data | 1987 |
| Rio Areia | Paraná | South | 1,395.95 | Tenured | 1996 | 1998 |
| Rio Branco Itanhaém | São Paulo | Southeast | 2,846.37 | Tenured | No data | 1987 |
| Rio das Cobras | Paraná | South | 19,017.31 | Tenured | No data | 1991 |
| Rio dos Pardos | Santa Catarina | South | 744.02 | Tenured | 1993 | 2000 |
| São Jerônimo | Paraná | South | 1,359.37 | Tenured | No data | 1991 |
| Serra do Itatins | São Paulo | Southeast | 1,204.59 | Tenured | No data | 1987 |
| Sete Cerros | Mato Grosso do Sul | Center-West | 8,984.08 | Tenured | 1991 | 1993 |
| Takuaraty/Yvykuarusu | Mato Grosso do Sul | Center-West | 2,590.05 | Tenured | 1992 | 1993 |
| Arroio-Korá | Mato Grosso do Sul | Center-West | 7,126.59 | Tenured | 2006 | 2009 |
| Tibagy/Mococa | Paraná | South | 844.35 | Tenured | No data | 1996 |
| Toldo Chimbangue | Santa Catarina | South | 1,003.71 | Tenured | No data | 1991 |
| Toldo Chimbangue II | Santa Catarina | South | 962.7 | Tenured | 2002 | 2006 |
| Vanuíre | São Paulo | Southeast | 717.22 | Tenured | No data | 1991 |
| Varzinha | Rio Grande do Sul | South | 772.35 | Tenured | 2001 | 2003 |
| Ventarra | Rio Grande do Sul | South | 741.99 | Tenured | 1996 | 1998 |
| Votouro | Rio Grande do Sul | South | 3,357.29 | Tenured | No data | 2000 |
| Águas Belas | Bahia | Northeast | 1,197.99 | Tenured | 1996 | 1998 |
| Xapecó | Santa Catarina | South | 15,703.82 | Tenured | No data | 1991 |
| Barão de Antonina | Paraná | South | 3,774.43 | Tenured | No data | 1991 |
| Barra Velha | Bahia | Northeast | 9,033.68 | Tenured | No data | 1991 |
| Caieiras Velha II | Espírito Santo | Southeast | 60.35 | Tenured | 2002 | 2004 |
| Boa Vista Sertão do Promirim | São Paulo | Southeast | 947.21 | Tenured | No data | 2000 |
| Caarapó | Mato Grosso do Sul | Center-West | 3,613.49 | Tenured | No data | 1991 |
| Cacique Doble | Rio Grande do Sul | South | 4,417.49 | Tenured | No data | 1991 |
| Tupiniquim | Espírito Santo | Southeast | 14,264.47 | Tenured | 2007 | 2010 |
| Aldeia Limão Verde | Mato Grosso do Sul | Center-West | 684.52 | Tenured | No data | 1928 |
| Carreteiro | Rio Grande do Sul | South | 609.81 | Tenured | No data | 1991 |
| Cerrito | Mato Grosso do Sul | Center-West | 2,016.55 | Tenured | 1991 | 1992 |
| Guarani de Araçaí | Santa Catarina | South | 2.724.32 | Declared | 2007 | N/A |
| Guyraroká | Mato Grosso do Sul | Center-West | 11,289.65 | Declared | 2009 | N/A |
| Ibirama La Klanô | Santa Catarina | South | 36,888.32 | Declared | 2003 | N/A |
| Itaóca | São Paulo | Southeast | 547.41 | Declared | 2000 | N/A |
| Jaraguá | São Paulo | Southeast | 532.38 | Declared | 2015 | N/A |
| Kariri-Xocó | Alagoas | Northeast | 4,677.66 | Declared | 2006 | N/A |
| Tenondé Porã | São Paulo | Southeast | 15,917.69 | Declared | 2016 | N/A |
| Morro Alto | Santa Catarina | South | 887.23 | Declared | 2009 | N/A |
| Morro dos Cavalos | Santa Catarina | South | 1993 | Declared | 2008 | N/A |
| Toldo Pinhal | Santa Catarina | South | 4,841.66 | Declared | 2007 | N/A |
| Piraí | Santa Catarina | South | 3,001.64 | Declared | 2009 | N/A |
| Yvy-Katu | Mato Grosso do Sul | Center-West | 9,477.32 | Declared | 2005 | N/A |
| Potiguara de Monte-Mor | Paraíba | Northeast | 7,258.88 | Declared | 2007 | N/A |
| Potrero Guaçu | Mato Grosso do Sul | Center-West | 4,050.11 | Declared | 2000 | N/A |
| Ribeirão Silveira | São Paulo | Southeast | 8,465.94 | Declared | 2008 | N/A |
| Rio dos Índios | Rio Grande do Sul | South | 733.2 | Declared | 2004 | N/A |
| Taquara | Mato Grosso do Sul | Center-West | 9,591.72 | Declared | 2010 | N/A |
| Pindoty | Santa Catarina | South | 3,233.39 | Declared | 2010 | N/A |
| Toldo Imbu | Santa Catarina | South | 1,873.93 | Declared | 2007 | N/A |
| Wassu-Cocal | Alagoas | Northeast | 2,736.52 | Delimited | N/A | N/A |
| Herarekâ Xetá | Paraná | South | 2,680.48 | Delimited | N/A | N/A |
| Mato Preto | Rio Grande do Sul | South | 4,189.11 | Declared | 2012 | N/A |
| Votouro/Kandóia | Rio Grande do Sul | South | 5,958.56 | Delimited | N/A | N/A |
| Sombrerito | Mato Grosso do Sul | Center-West | 12,860.85 | Declared | 2010 | N/A |
| Tupinambá de Olivença | Bahia | Northeast | 47,415.7 | Delimited | N/A | N/A |
| Barra Velha do Monte Pascoal | Bahia | Northeast | 43,684.04 | Delimited | N/A | N/A |
| Boa Vista | Paraná | South | 6,568.83 | Declared | 2007 | N/A |
| Tarumã | Santa Catarina | South | 2,197.97 | Declared | 2009 | N/A |
| Boa Vista Sertão do Promirim | São Paulo | Southeast | 5,458.46 | Delimited | N/A | N/A |
| Yvyporã Laranjinha | Paraná | South | 1,262.23 | Declared | 2007 | N/A |
| Panambi - Lagoa Rica | Mato Grosso do Sul | Center-West | 12,119.39 | Delimited | N/A | N/A |
| Xapecó (Pinhalzinho-Canhadão) | Santa Catarina | South | 621.11 | Declared | 2004 | N/A |
| Comexatibá | Bahia | Northeast | 28,023.09 | Delimited | N/A | N/A |
| Passo Grande do Rio Forquilha | Rio Grande do Sul | South | 1,893.9 | Declared | 2011 | N/A |
| Tekohá Jevy (Rio Pequeno) | Rio de Janeiro | Southeast | 2,281.01 | Delimited | N/A | N/A |
| Dourados-Amambaipeguá I | Mato Grosso do Sul | Center-West | 55,210.06 | Delimited | N/A | N/A |
| Cerco Grande | Paraná | South | 1,418.85 | Delimited | N/A | N/A |
| Sambaqui | Paraná | South | 2,885.83 | Delimited | N/A | N/A |
| Mato Castelhano | Rio Grande do Sul | South | 3,483.07 | Delimited | N/A | N/A |
| Tupinambá de Belmonte | Bahia | Northeast | 9,602.39 | Delimited | N/A | N/A |
| Pindoty / Araçá-Mirim (Tekohá Pindoty) | São Paulo | Southeast | 1,020.79 | Delimited | N/A | N/A |
| Tapy'i/Rio Branquinho | São Paulo | Southeast | 1,144.53 | Delimited | N/A | N/A |
| Guaviraty | São Paulo | Southeast | 1,185.11 | Delimited | N/A | N/A |
| Ka'aguy Mirim | São Paulo | Southeast | 1,167.93 | Delimited | N/A | N/A |
| Djaiko-Aty | São Paulo | Southeast | 1,205.58 | Delimited | N/A | N/A |
| Amba Porã | São Paulo | Southeast | 7,088.59 | Delimited | N/A | N/A |
| Peguaoty | São Paulo | Southeast | 6,146.53 | Delimited | N/A | N/A |
| Pakurity (Ilha do Cardoso) | São Paulo | Southeast | 5,657.02 | Delimited | N/A | N/A |
| Ka'aguy Hovy (Vale do Ribeira II) | São Paulo | Southeast | 1,963.22 | Delimited | N/A | N/A |
| Iguatemipegua I | Mato Grosso do Sul | Center-West | 41,580.64 | Delimited | N/A | N/A |
| Ypoi / Triunfo | Mato Grosso do Sul | Center-West | 19,960.88 | Delimited | N/A | N/A |
| Aldeia Velha | Bahia | Northeast | 2,025.63 | Declared | 2011 | N/A |
| N/A values represent data that does not exist because Indigenous lands have not been tenured and/or declared. | | | | | | |
| Regions of Brazil were sourced from: <https://www.ibge.gov.br/geociencias/cartas-e-mapas/mapas-regionais/10861-mapas-regionais.html?=&t=acesso-ao-produto> | | | | | | |
| The total area of Indigenous lands in this study is 7.45*10^5^ ha, which was approximately 0.67% of the 1.11*10^8^ ha of the entire Atlantic Forest biome. | | | | | | |

**SI References**

1. C. Araujo, C. A. Bonjean, J.-L. Combes, P. Combes Motel, E. J. Reis, Property rights and deforestation in the Brazilian Amazon. *Ecol. Econ.* **68**, 2461–2468 (2009).

2. M. T. Buntaine, S. E. Hamilton, M. Millones, Titling community land to prevent deforestation: An evaluation of a best-case program in Morona-Santiago, Ecuador. *Glob. Environ. Change* **33**, 32–43 (2015).

3. A. BenYishay, S. Heuser, D. Runfola, R. Trichler, Indigenous land rights and deforestation: Evidence from the Brazilian Amazon. *J. Environ. Econ. Manag.* **86**, 29–47 (2017).

4. L. C. Barbosa, *The Brazilian Amazon Rainforest: Global Ecopolitics, Development, and Democracy* (University Press of America, 2000).

5. S. Wiessner, Rights and Status of Indigenous Peoples: A Global Comparative and International Legal Analysis. *Harv. Hum. Rights J.* **12**, 57–128 (1999).

6. A. L. V. Nötzold, S. F. Bringmann, O Serviço de Proteção aos Índios e os projetos de desenvolvimento dos Postos Indígenas: o Programa Pecuário e a Campanha do Trigo entre os Kaingang da IR7. *Rev. Bras. História Ciênc. Sociais* **5** (2013).

7. C. S. de Almeida, A. L. V. Nötzold, ENTRE O “MATO VIRGEM” E OS “NEGÓCIOS DA MADEIRA”: CAMINHOS DO INDIGENISMO BRASILEIRO EM TERRAS KAINGANG. *Mov. Sociais E Resist. No Sul Bras.* (2021).

8. R. Fernandes, L. Piovezana, The Kaingang perspectives on land and environmental rights in the south of Brazil. *Ambiente Soc.* **18**, 111–128 (2015).

9. A. Borges, Arrendamento ilegal de terras indígenas compromete 3,1 milhões de hectares. *Estadão* (2018) (March 15, 2022).

10. K. V. Conceição, *et al.*, Government policies endanger the indigenous peoples of the Brazilian Amazon. *Land Use Policy* **108**, 105663 (2021).

11. S. Rodrigues-Filho, *et al.*, Election-driven weakening of deforestation control in the Brazilian Amazon. *Land Use Policy* **43**, 111–118 (2015).

12. Cacique e lideranças indígenas são condenados por arrendamento de terras em Nonoai. *G1* (2019) (March 14, 2022).

13. Milícias armadas criadas por caciques espalham o terror em aldeias indígenas no Rio Grande do Sul. *G1* (2021) (March 15, 2022).

14. F. E. T. Cancela, História dos Pataxó no Extremo Sul da Bahia. *Abatirá-Rev. Ciênc. Humanas E Linguagens* **1**, 18–49 (2020).

15. A. Blackman, L. Corral, E. S. Lima, G. P. Asner, Titling indigenous communities protects forests in the Peruvian Amazon. *Proc. Natl. Acad. Sci.* **114**, 4123–4128 (2017).

16. K. Baragwanath, E. Bayi, Collective property rights reduce deforestation in the Brazilian Amazon. *Proc. Natl. Acad. Sci.* **117**, 20495–20502 (2020).

17. B. Callaway, P. H. C. Sant’Anna, Difference-in-Differences with multiple time periods. *J. Econom.* **225**, 200–230 (2021).

18. C. De Chaisemartin, X. d’Haultfoeuille, Two-way fixed effects estimators with heterogeneous treatment effects. *Am. Econ. Rev.* **110**, 2964–96 (2020).

19. L. Sun, S. Abraham, Estimating dynamic treatment effects in event studies with heterogeneous treatment effects. *J. Econom.* **225**, 175–199 (2021).

20. K. Imai, I. S. Kim, On the use of two-way fixed effects regression models for causal inference with panel data. *Polit. Anal.* **29**, 405–415 (2021).

21. Terras Indígenas no Brasil (2022) (February 6, 2022).
